# Linking work characteristics to proactive behavior: Mediating role of motivational state

**DOI:** 10.1016/j.heliyon.2023.e17522

**Published:** 2023-06-28

**Authors:** Woretaw Chanie, Solomon Melese, Assegid Demesie

**Affiliations:** a Department of Management, College of Business and Economics, University of Gondar, Ethiopia; b College of Business and Economics, University of Gondar, Ethiopia

**Keywords:** Contextual characteristics, Motivational state, Proactive behavior, Knowledge characteristics, Social characteristics, And task characteristics

## Abstract

**Purpose:**

The fascinating topic of work design was demonstrated by academics using work characteristics. There is still much to learn about proactive behavior in contemporary organizations. In this study, proactive behavior was linked to workplace features using a variety of metrics and a motivational state as a mediator.

**Methodology:**

In order to accomplish this, survey research was the writers' favored technique. 279 respondents provided self-reporting information and supervisory assessments, from which we acquired data. The analysis was done using the SMART PLS software.

**Conclusion:**

The authors arrive at this conclusion after determining that task, knowledge, and contextual factors were most likely linked to proactive employee behavior; social traits and proactive behavior, however, were indirectly related. When it comes to the association between work qualities (task, knowledge, and contextual factors), the motivated state only partially mediates the relationship while it entirely mediates the relationship between social features and proactive behavior. We encourage further investigation to bolster our findings and broaden our model by discovering additional employee outcomes connected to pro-active behavior and social traits.

**Originality/value:**

The whole relationship between work characteristics and proactive behavior had not been fully explored in other studies. As a result, our research made tremendous progress in our knowledge of how workplace traits and a variety of proactive behaviors connect to motivation.

## 1 Introduction

Numerous studies that looked at the implementation and results of changes in work and job structure, as well as individual, group, and organizational performance, have been conducted [1]. Hackman and Oldham [2] presented a model of job characteristics with five distinctive core job aspects and three crucial psychological states as the foundation for theoretical models of work design. It is currently believed that these five fundamental work qualities are too restricted to apply to all occupations [3,4]. In order to more fully describe employment, the five job criteria were eventually expanded with other labor qualities. According to Cai, Parker, Chen, and Lam [5] and Parker, Wall, and Cordery [6], knowledge, social, and contextual considerations have been added to the primary job requirement (task).

Grant, Fried, and Juillerat [7] and De Stobbeleir, Ashford, and Zhang (8), first identified the connection between task characteristics and the kind and variety of tasks performed in employment as well as how the job is carried out. While knowledge qualities show the skill and ability needed by a body for the jobs it completes [[7], [8], [9]]. Grant, Fried, and Juillerat [7] also connected social distinctiveness to the number and nature of interpersonal connections that employees must preserve at work. They continued by stating that this is the case because they connect the uniqueness of the working framework to the employee's surroundings and the tasks that must be completed.

Proactive behavior was suggested by the four aspects of the work characteristics [9,10]. In their adaptation of the work design theory, Grant and Parker [1] established a “dynamic model” of proactive behavior and job design. They backed two essential ideas, “relational” and “proactive,” in order to account for workplace traits and proactive behavior. The former focuses on how tasks, roles, and jobs are more firmly defined than ever, while the later emphasizes the encouraging early results of employee-pleasing initiatives that aim to foresee and influence future changes in how work is carried out. It's crucial to connect proactive behavior to the four components of work characteristics.

The association between these work traits and proactive behavior was further explained by the motivational state [4]. This idea was explained using both theoretical and empirical data. Grant and Parker's [1] dynamic model of proactive behavior and job design with a motivational state linking mechanism made perfect sense theoretically. The task, knowledge, social, and contextual elements of the workplace can be used by the motivational state to perceive proactive behavior, according to empirical research by Ohly and Fritz [11]. As a result, the motivated state provides a useful theoretical framework for examining mediating elements that might link personality qualities connected to the workplace to proactive behavior.

In today's workplaces, which are marked by rapid change, little monitoring, intense competition, and high uncertainty [10,12,13], proactive behavior is essential. People must be proactive at work due to the uncertainty and constant change in the business environment [14,15], and organizations profit from proactive employees who make improvements straight away [5]. As a result, research on corporate proactivity will continue to be interesting and significant soon [16].

The association between proactive behavior and workplace traits has been the subject of previous theoretical and experimental studies. Crant [12], a pioneer of proactive behavior in organizations, was the first to identify its distinguishing characteristics, and researchers were developing the full range of labor skills [6,9]. In addition to Grant & Parker's (1) thorough introduction of the redesigning of the work design theory, which demonstrated a direct correlation between work characteristics and proactive conduct, (10) introduced three higher-order proactive behaviors with measurement issues related to the work characteristics. Empirical studies [such as Refs. (5, 7, 8, 14, 15, and 16]] gave details on proactive behavior and work characteristics in organizations with certain dimensions.

Although the authors discussed the significant contribution of past studies, there were empirical, methodological, and contextual concerns that needed to be addressed in order to support this study.

Empirically, a particular proactive behavior trait was positively associated with a particular set of professional traits. However, they did not completely understand the causes of and consequences of proactive behavior [17,18]. In different places, neither the direct nor indirect connections by themselves were statistically significant. Job autonomy (task characteristics) is an example of an indirect link that explains proactive behavior in the context of the mediation processes of perceived behavioral control and intent, work engagement [19], and perceived behavioral control [17]. Although there was a direct correlation between proactive work behavior and job autonomy [14], proactive behavior among employees also showed a relationship with social context features [5]. However, past studies [3,20] only used one set of proactive behavior as an outcome variable and [18] did not fully link work characteristics and proactive behavior through a motivational state (energizing state). A clear picture of proactive behavior should not exist during this motivational (energizing) state [10]. So, using the energized state as a mediating mechanism, the authors addressed the full relationship between work place trait and proactive behavior.

The work characteristics and proactivity investigations conducted by earlier researchers [e.g., Refs. [8,10,21,and22]]] employed a self-reported measure as part of their approach. The “self-reported” proactive conduct measure does have another special drawback, though, in that we cannot be certain that observers would spot the same behavioral differences [10]. The task, knowledge, social and contextual work characteristics as well as proactive behavior variables have respective second- and third-order constructs as sub-dimensions. Numerous studies measured these constructs as first-order constructs. Thus, it is essential to consider acquiring relevant data from multiple sources utilizing a variety of data collection technologies and measuring as second- and third-order constructs when evaluating work characteristics and proactive behavior [4].

To compete globally, organizations must contextually enhance their operational and administrative processes [12]. Improve product cost, time to market, and quality, for instance Refs. [22,23], in sectors supported by proactive strategies. Industries must acknowledge and comprehend employees' behaviors and feelings in order to establish a workplace where proactive people will be helpful [5]. There haven't been any studies in this area, particularly in Ethiopia.

This study was distinct from others in that it established a full association between work characteristics and proactive behavior along with a distinct picture of the motivational state (energized to state), which is how this study was different from them. The authors used two assessments (the self-report and supervisor ratings) in Ethiopian industrial parks and also considered task, knowledge, social, and contextual aspects as second-order constructs and employees' proactive behavior as a third-order latent construct.

This research made a substantial contribution to the thorough investigation of how the motivated state links workplace features to a variety of proactive behaviors. To promote employee proactiveness in industrial parks, it also ensures that proactive behavior is practically connected with task, knowledge, social, and contextual features. Utilizing the motivational state as a mediator in the context of industrial environments, the current study's goal is to investigate the relationship between proactive behavior and work-related features.

## 2 Theoretical framework

Work design significantly influenced employee attitudes, behaviors, and well-being [6,15,24], which is linked to four work characteristics [1]. With the energized state acting as a connecting mechanism, we have discovered a relationship between these four work qualities and proactive behavior. Such an approach has been used to explore various hypotheses.

Although there are changes taking place in work design theory and research [1], a number of theories, including job design theory, affective events theory, planned behavior theory, and proactive motivational state theory, were established as the underlying principles that connected work features and proactive behavior.

Understanding when and how specific job designs affect employee outcomes, particularly proactive behavior, is a key goal of the field of job design theory [25]. This is demonstrated because affective events theory offers a helpful framework for conceptualizing and evaluating the underlying mechanisms fundamental the association between work qualities and proactive behavior [26]. The theory of planned behavior also looked at key preconditions and mental processes that underlie proactive behavior in professional settings [27]. In contrast, the idea of proactive motivational state claimed that motivational states were reliable and effective predictors of proactive behavior [10]. As a result, the relationship between workplace characteristics and proactive behavior as well as the mediating function of motivated state has been described as a head.

### 2.1 Linking work characteristics to proactive behavior

In-depth study has connected specific work qualities to proactive behavior dimensions, despite the fact that theoretical models indicate that work characteristics will lead to proactive behavior [28]. For instance, proactive behavior is predicted by job autonomy [3] as well as career-related behavior [26,27] and proactive behavior at work [3]. In addition to confirming the relationship between task interdependence and proactive person-environment fit behavior (peer feedback-seeking), De Stobbeleir, Ashford, and Zhang [8] also confirmed that proactive behavior was task-dependent [29].

Individual knowledge attributes have an impact on employee proactivity. In light of this, “employees with preferences for high levels of knowledge characteristics enjoy applying their unique knowledge and skills to ambiguous and uncertain work tasks” [28] p987]. Higher knowledge employees are more inclined to engage in proactive behavior. Thus, knowledge serves as the basis for proactive behavior [4]. The relationship between knowledge traits and proactive behavior was also established by Dust and Resick [29].

An interactionist view of proactive behavior would presuppose that “social context shapes individual decisions to be proactive” [5 p1]. Employees should therefore provide an example of proactive behavior when working with colleagues. According to various academics, social context variables were crucial for promoting proactive behavior [30], social support, and collective proactive behavior [5]. Furthermore, “social context shapes individual decisions to be proactive” [5 p1]. Social factors in this case directly influence proactive behavior.

The physical and environmental surroundings of the worker and the tasks that must be completed are related to work context characteristics. The antecedents of proactive behavior have been identified as contextual characteristics (leadership, interpersonal climate, and social processes) [4]. This demonstrates how context can help employees who are acting pro-actively achieve great results. This is why they work to change their context [31] and their job resources (equipment use) [32]. So, we suggest:

**Ha**_**1:**_ There is a significant positive linkage between task characteristics and proactive behavior.

**Ha**_**2**_: There is a significant positive linkage between knowledge characteristics and proactive behavior.

**Ha**_**3:**_ There is a significant positive linkage between social characteristics and proactive behavior.

**Ha**_**4:**_ There is a significant positive linkage between contextual characteristics and proactive behavior.

### 2.2 The link between work characteristics and motivational state

According to the notion of purposeful work behavior, every job has both social and task features, which define factors that influence motivation at work [33]. The awareness that task, knowledge, social, and environmental variables are likely to have an impact on the efficacy of job design has therefore advanced [4].

Variables related to work qualities could theoretically be connected to motivated state. It follows that “aspects of the work context can intervene to prevent individuals high can do, reason to, and energized to motivations from being proactive” [4 p840]. Job enrichment (job features) is also likely to have an impact on reasons for motivation, leading to more ‘energized’ motivation in workers [4,32]. Hence, we suggest;

**Ha**_**5:**_ There is a significant positive linkage between task characteristics and motivational state.

Ha6: There is a significant positive linkage between knowledge characteristics and motivational state.

Ha7: There is a significant positive linkage between social characteristics and motivational state.

Ha8: There is a significant positive linkage between contextual characteristics and motivational state.

### 2.3 The association between motivational state and proactive behavior

Recently, the concept of being “energized to state” has gained popularity [34] and has been shown to encourage proactive behavior [4]. It is crucial for encouraging proactive work behavior among employees. According to several theories, positive affect is a “hot” psychological power that reliably predicts initiative. For instance, researchers put forth the neuropsychological notion that good affect boosts people's capacity for different cognitive perspectives and predicts problem-solving (proactive work behavior) [35]. Additionally, according to the notion of motivational state, feeling energized is a reliable predictor of proactive behavior [4]. Therefore, we suggest that:

**Ha**_**9**_: There is a significant positive linkage between motivational state and proactive behavior.

### 2.4 Mediating role of motivational state between work characteristics and proactive behavior

There is substantial evidence that the motivational state's cause influences the association between work traits and proactive behavior. Energized to state, for example, is “the key direct positive affect pathway influencing goal generation and striving across a range of proactive goals” [4 p840] and develops and broadens an individual's physical, intellectual, and social resources, which may help initiate effortful and goal-oriented behaviors, such as proactive behavior, because when employees are in a positive affective state, they are more likely to set challenging goals and engage in proactive goal generation.

The researchers also verified the inverse association between proactive behavior and task features. When perceived behavioral control and intentions were mediated, Shin and Kim [17] responded that job autonomy impacts proactive behavior indirectly. Social setting indirectly promotes proactive work behavior, according to Cai, Parker, Chen, and Lam [5]. Along with Sonnentag [36], Wang, Xu, Sun, and Liu [37] discovered that pro-social effect and proactive work behavior were both mediated by a motivational state and that perceived pro-social impact predicted both positive affect (energize to) and those situational constraints. Hence, we suggest;

**Ha**_**10**:_ Motivational state mediates the link between task characteristics and proactive behavior.

Ha11: Motivational state mediates the link between knowledge characteristics and proactive behavior.

Ha12: Motivational state mediates the link between social characteristics and proactive behavior.

Ha13: Motivational state mediates the link between contextual characteristics and proactive behavior.

**Fig. 1:**
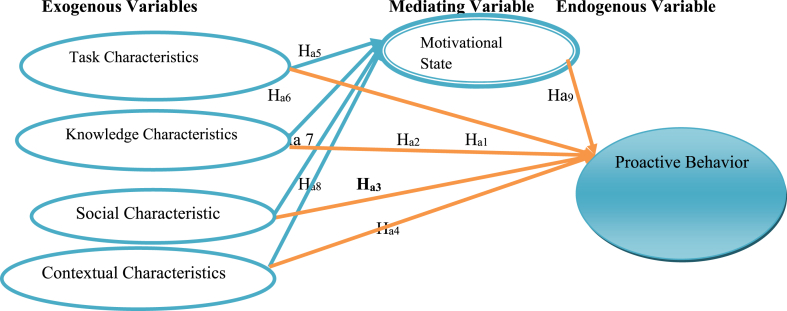
Hypothesized conceptual frame work of the study.

Source: adapted from (Morgeson & Humphrey 2006; Parker & Bindl 2016; 2010, Parker & Collins, 2010)

Generally, authors developed a conceptual framework of the study in Fig. 1.

## 3 Methodology

### 3.1 Research design

The authors aim to examine the link between work characteristics and proactive behavior by taking the motivational state as a mediator by gathering information from a sample of respondents with survey questionnaires. Thus, the authors used quantitative research choices with a cross-sectional survey strategy.

### 3.2 Sample and procedures

The authors focused the study on three active sites in industrial parks in Ethiopia with garment enterprises due to difficulties paired with geographically separated populations where face-to-face contact is necessary or if it is luxury and lengthy [38]. The authors concentrated on industries with garment companies because the working conditions in industrial parks vary depending on the industry. We hypothesized that these businesses manufacture roughly identical goods and had comparable work cultures. Using purposive sampling methods, the industrial parks in Hawassa, Bole Leme, and Debre Berhan were chosen.

Researchers used several raters and chose the employees and supervisors using a straightforward random sampling procedure in order to study adequate information and address frequent method bias. In order to create a sampling frame for a wide geographic area, this study focused on 26381 operational level employees and supervisors from 11 organizations. The formula Yamane presented to determine sample size was simplified, and it is still the one that is most frequently used today. This leads us to conclude that the study's sample size should be 394 respondents (197 employees and 197 supervisors) from the study's target population.

### 3.3 Instrument

The researchers used close-ended questionnaires. The main database for realizing the research objectives and testing the hypothesis is to be fetched through the index, which would be as carefully structured, self-administered, and supervisory-measured questionnaires. Different questionnaires (INDEX) were prepared and administered for each of the primary sources. In industrial parks, the task, knowledge, social, and contextual characteristics items, as well as proactive behavior items that are believed to be judged by employees and supervisors, would be lumped together, forming the Employee Questionnaire (INDEX), and Supervisor Questionnaire (INDEX). The number and the concepts of all the items were the same. The only difference was the subject in which a self-report questionnaire was formed as “I--" while we structured the supervisor ratings as “S/He--". Supervisors rated employees on the same work characteristics and proactive behavior scales as were completed by employees. We received 279 questionnaires from samples of employees (#146) and their immediate supervisors (#133). We secured ethical approval from the Ethics Committee of UoG Business and Economics College, the School of Management, and the public administration research committee, and we got informed consent from all patients/participants for our survey.

#### 3.3.1 Work characteristics

The “Work Design Questionnaire (WDQ) measured work characteristics” [9], contains four major dimensions, and 18 sub-dimensions, namely task characteristics with 5 sub-dimensions (14 items), knowledge characteristics with 5 sub-dimensions (12 items), social characteristics with 4 sub-dimensions (12 items), and contextual characteristics with 4 sub-dimensions (14 items), for 52 items, with a 5-point Likert scale from strongly disagree (1) to strongly agree (5), adopted from García-izquierdo, and Castaño [39], a “shortened version” of the Work Design Questionnaire (WDQ). It was first developed by Morgeson and Humphrey [40] and validated by Ríos, Vielma, Carlos, García, Aravena, and David [41]. Both a self-report measure and a supervisory measure of work characteristics would be considered. Because the “work design questionnaire (WDQ) appears to hold promise as a general measure of work characteristics that can be used by scholars and practitioners to conduct basic research on work or to design and redesign jobs in organizations” [39] p206], a reliable and valid measure of work characteristics with patent prospective applications in research [41].

#### 3.3.2 Proactive behavior

The authors measured proactive behavior as a third-order construct with 25 items, having five Likert scales adopted [10]. We considered both a self-reported measure and a supervisory measure of proactive behavior. Explicitly, proactive work behavior would be measured with voice, taking charge, individual innovation, and problem prevention having 13 items with a Likert scale ranging from very infrequently (1) to very frequently (5). While proactive person-environment fit behavior would be measured with a 12-item scale containing feedback monitoring and feedback inquiry having a response scale ranging from infrequently (1) to very frequently (5); job change negotiation having a response scale from to no extent (1) to a great extent (5); and career initiative having a response scale from strongly disagree (1) to strongly agree (5), because higher-order proactive behavior categories examine similarities, differences, and interrelationships surrounded by various types of proactive behavior [10].

#### 3.3.3 Motivational state

The energized state explained the motivational state because it is a hot state, which leads to more employees being proactive. We measure energized state with positive affect at work in which subjective vitality is how measures positive affect using Ryan and Frederick's [42] seven-item measure having a 5-point Likert scale from not at all true (1) to very true (5), adding the phrase at work to the end of each item. Castillo, Tomás, and Balaguer [43] validated these items. The reason the researchers chose subjective vitality as a measure of positive affect is first, subjective vitality is conceptually similar to the feeling of positive affect [43]. In addition, positive affects [43] such as excitement, enthusiasm, and energy are likely the same as the subjective vitality scale, like “I have energy and spirit at work”.

## 4 Results

### 4.1 Descriptive statistics

On July 20, 2022, the researchers delivered 394 questionnaires (to 201 employees and 193 supervisors), and 299 responses (from 159 employees and 140 supervisors) were received, for a return rate of 75.88%. 279 (93.35%) of the surveys that were returned could be used; the remaining surveys, including those that had over 15% of their items missing, were improperly filed and unusable.

The demographic details of the respondents were analyzed following our survey. In terms of educational attainment, out of 279 respondents, 167 (57.9%) had a diploma (99 employees and 67 supervisors). Out of 279 responders, the majority of 245 (87.8%) had 0 to 5 years of job experience. Respondents no longer encounter industrial parks because they are arriving earlier in Ethiopia.

The descriptive statistics for task, knowledge, social, and contextual characteristics show an overall mean score of 3.429 (SD = 0.801), 3.492 (SD = 0.813), 3.494 (SD = 0.813), and 3.55 (SD = 0.773), respectively. This indicates the extent to which employees moderately exercise tasks, knowledge, social and contextual characteristics within industrial parks. The overall motivational state and proactive behavior also show a mean score of 3.450 (SD = 0.777) and 3.456 (SD = 0.967), respectively. This shows the extent to which employees' motivational states and proactive behaviors were somewhat good in industrial parks, but they required more energy to engage proactively in their work.

### 4.2 Inferential statistics: model evaluation

This study used PLS-SEM and considered both the measurement (outer) and structural (inner) models. The author described the underlying assumptions of PLS-SEM based on these two models using higher-order constructs. It has become easier to represent a construct on a more abstract higher-level dimension (higher-order component) and its more concrete lower-order subdimensions as a result of the use of higher-order constructs in PLS-SEM applications [44]. Higher-order latent components can be expressed in four distinct ways [45], however, for this investigation, the reflective formative model with repeated indications was adopted. Due to its widespread adoption [46], which also leads to a less biassed relationship between higher- and lower-order components [45], this strategy is the most widely employed.

The test for normality of distribution is not problematic in PLS-SEM when the research is focused on complex theoretical models with a large number of indicators, different endogenous and exogenous components, or non-normal statistical distributions [45]. This study did not do a test for normality as a result.

#### 4.2.1 Measurement models

The authors used SMART PLS 3 while running the model. The SMART PLS method developed by Hair, Hult, and Ringle [47] provides an explanation for the general quality of the measurement model. In this study, the SMART-PLS algorithm was used. A reflective-formative measurement model with a path weighting scheme and a cap of 300 iterations with a stop condition of 7 were utilized. It is important for researchers to confirm the first-order constructs' “reliability” and “validity” [47], taking into account the four criteria of (i) factor loadings, (ii) indicator reliability, (iii) convergent validity, and (iv) discriminate validity.

##### 4.2.1.1 Factor loadings

Prior to determining an indicator's “reliability” and “validity,” the researchers look at its loadings in the first model. According to Hair, Sarstedt, Ringle, and Mena [46], loadings should be higher than 0.70. Furthermore, Hair, Hult, and Ringle [47] showed that in order to offer satisfactory item reliability, the constructs must account for more than 50% of the variance in the indicator.

As the authors showed, the outer loadings of the drafted model, which consisted of one motivational state item and eight items linked to work-related attributes, were loaded less than 0.5. Outer loads of other components ranged from 0.765 to 0.919. This shows that the structure accounts for more than 50% of the indicator's variation [47], demonstrating acceptable item reliability.

**Fig. 2:**
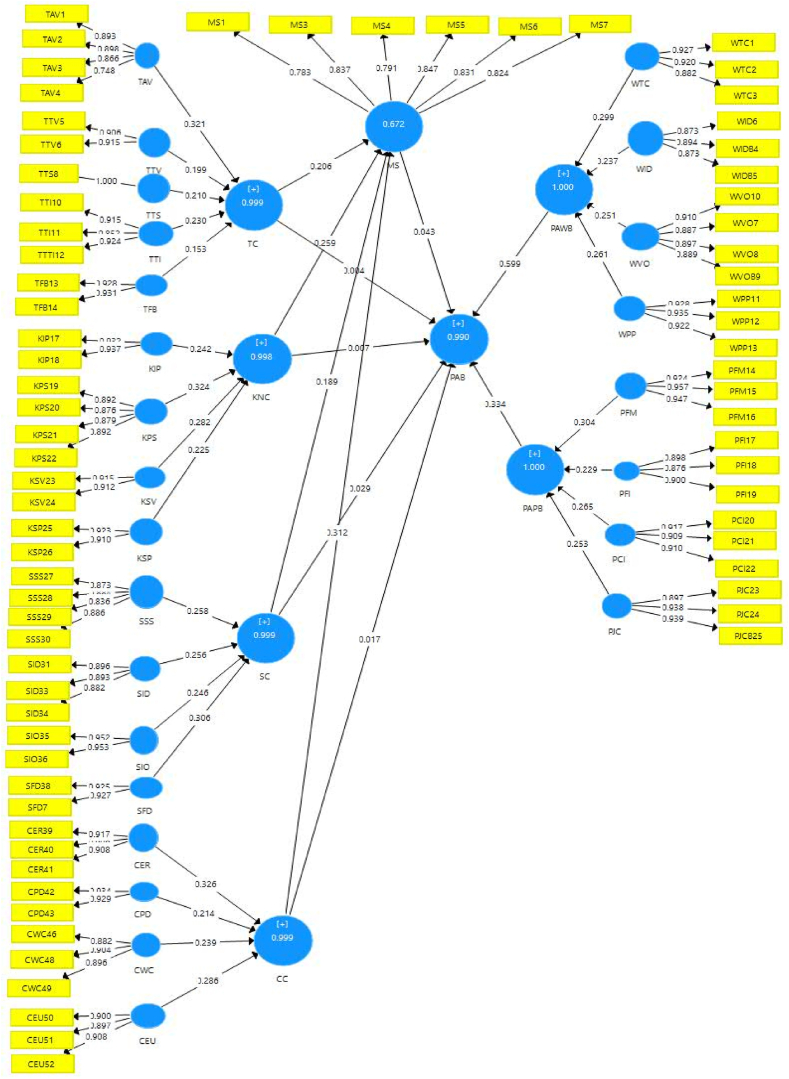
Final path model.

Later, to enhance model fit, the dimension nine items that supported the latent constructs that were irrelevant to the model were eliminated. The final path model depicted in Fig. 2 was developed as a result of the research process, and the authors should use the model evaluation to improve the validity and dependability of their work. We, therefore, verify first-order, second-order, and third-order structures.

##### 4.2.1.2 Reliability and validity analysis

In Table 1, the constructs' “average variance extracted” and “composite reliability” are shown. Conducting two important tests is indicated in order to assess the validity and dependability of measuring procedures [48]. The two reliability test methods that are most frequently used are Cronbach's Alpha and composite reliability. Table 1 shows values for both Cronbach's Alpha and composite reliability that are both significantly higher than 0.7. Reliability levels greater than 0.7 are present in all first-order constructions [46]. This establishes construct reliability.

**Table 1:** Construct reliability and validity.

Constructs			Cronbach's Alpha	Composite Reliability	Average Variance Extracted (AVE)
Third Order	Second order	First order			
–	TC	TAV	0.874	0.914	0.729
		TFB	0.842	0.927	0.863
		TTI	0.879	0.925	0.805
		TTV	0.795	0.907	0.830
	KNC	KPS	0.907	0.935	0.783
		KSP	0.810	0.913	0.840
		KSV	0.801	0.909	0.834
		KIP	0.855	0.932	0.873
	SC	SFD	0.834	0.923	0.857
		SID	0.869	0.920	0.793
		SIO	0.897	0.951	0.907
		SSS	0.893	0.926	0.757
	CC	CER	0.896	0.935	0.828
		CEU	0.885	0.929	0.813
		CPD	0.847	0.929	0.867
		CWC	0.874	0.923	0.799
PAB	PAWB	WID	0.855	0.912	0.775
		WPP	0.920	0.949	0.861
		WTC	0.896	0.935	0.828
		WVO	0.918	0.942	0.803
	PAPB	PFI	0.871	0.921	0.795
		PFM	0.937	0.960	0.889
		PJC	0.915	0.946	0.855
–	–	MS	0.902	0.924	0.671

Note. CC= Contextual characteristics; KNC= Knowledge Characteristics; MS= Motivational States; PAB= Proactive Behavior; PAWB= Proactive Work Behavior; PAPB= Proactive Person Environment Fit Behavior; SC= Social Characteristics and TC= Task Characteristics.

The variable loadings on the construct and an evaluation of redundancy are reflected in the convergent validity (AVE), a measure of validity in PLS-SEM [49]. It follows that average variance extracted (AVE) is used to assess convergent validity [46] and is considered acceptable when AVE is 0.50 or above, indicating that at least 50% of the variance of its products is explained by the construct [50]. The AVE test's results (Table 1) proved that the scores are higher than 0.67 or fall between 0.671 and 1.00. As a result, convergence validity was verified.

The Fornell-Larcker criterion and the heterotrait-monotrait (HTMT) ratio were used to assess discriminate validity. According to the Fornell-Larcker criterion, each construct's AVE should be greater than its squared correlation with any other construct, according to Franke, Sarstedt, and Franke [51]. Table 2 displayed the models for the “Fornell and Larcker” criterion test, which demonstrates appropriate discriminating validity because a bold diagonal value was higher than the value in its row and column.

**Table 2:** Fornell-Larcker criterion.

	CER	CEU	CPD	CWC	KIP	KPS	KSP	KSV	MS	PCI	PFI	PFM	PJC	SFD	SID	SIO	SSS	TAV	TFB	TTI	TTS	TTV	WID	WPP	WTC	WVO
CER	**0.910**																									
CEU	0.822	**0.902**																								
CPD	0.815	0.851	**0.931**																							
CWC	0.840	0.875	0.864	**0.894**																						
KIP	0.560	0.549	0.486	0.549	**0.934**																					
KPS	0.582	0.580	0.507	0.563	0.896	**0.885**																				
KSP	0.517	0.533	0.444	0.509	0.769	0.792	**0.917**																			
KSV	0.616	0.585	0.523	0.579	0.826	0.849	0.771	**0.913**																		
MS	0.705	0.686	0.665	0.676	0.634	0.664	0.595	0.638	**0.819**																	
PCI	0.691	0.688	0.647	0.670	0.668	0.683	0.615	0.660	0.789	**0.912**																
PFI	0.691	0.684	0.659	0.677	0.632	0.649	0.596	0.647	0.769	0.865	**0.892**															
PFM	0.724	0.706	0.651	0.691	0.719	0.747	0.662	0.726	0.790	0.876	0.879	**0.943**														
PJC	0.701	0.701	0.634	0.677	0.644	0.670	0.612	0.635	0.792	0.869	0.876	0.876	**0.925**													
SFD	0.603	0.661	0.611	0.634	0.515	0.538	0.453	0.490	0.627	0.608	0.577	0.584	0.591	**0.926**												
SID	0.596	0.631	0.613	0.600	0.464	0.467	0.372	0.423	0.626	0.580	0.538	0.531	0.558	0.843	**0.891**											
SIO	0.575	0.597	0.580	0.579	0.480	0.501	0.382	0.452	0.599	0.553	0.499	0.495	0.510	0.789	0.822	**0.952**										
SSS	0.594	0.644	0.603	0.610	0.460	0.489	0.388	0.436	0.637	0.568	0.540	0.548	0.559	0.866	0.882	0.827	**0.870**									
TAV	0.609	0.608	0.568	0.603	0.589	0.596	0.596	0.551	0.666	0.641	0.616	0.674	0.629	0.567	0.503	0.507	0.550	**0.854**								
TFB	0.523	0.476	0.448	0.457	0.491	0.538	0.473	0.502	0.529	0.518	0.476	0.525	0.464	0.533	0.467	0.458	0.540	0.712	**0.929**							
TTI	0.501	0.460	0.403	0.450	0.509	0.549	0.480	0.504	0.579	0.523	0.479	0.560	0.491	0.494	0.417	0.413	0.503	0.712	0.886	**0.897**						
TTS	0.513	0.475	0.426	0.468	0.493	0.522	0.481	0.495	0.568	0.477	0.477	0.529	0.491	0.516	0.437	0.423	0.497	0.690	0.829	0.836	**1.000**					
TTV	0.490	0.456	0.421	0.462	0.475	0.503	0.479	0.457	0.598	0.535	0.473	0.541	0.499	0.506	0.436	0.415	0.497	0.749	0.728	0.771	0.738	**0.911**				
WID	0.704	0.691	0.647	0.684	0.678	0.743	0.627	0.708	0.782	0.785	0.787	0.841	0.798	0.589	0.541	0.509	0.559	0.670	0.538	0.553	0.553	0.566	**0.880**			
WPP	0.724	0.719	0.671	0.697	0.708	0.732	0.649	0.721	0.767	0.868	0.873	0.921	0.869	0.585	0.530	0.509	0.534	0.673	0.530	0.542	0.499	0.524	0.864	**0.928**		

According to Murtala, Onukwube, and Yahaya's description of the heterotrait-monotrait (HTMT) correlation ratio, which is less than 1, this ratio can be used to investigate discrimination. As further indicated, bootstrapping can be used to determine whether the HTMT value significantly deviates from 1.00 or a lower threshold value. It showed no discriminating validity issues [52]. In light of the study's context, HTMT should therefore be defined [51]. Due to the fact that the HTMT value was less than 1, but slightly above 1, there were no problems with discriminating validity, as shown in Table 3.

**Table 3:** Hetrotrait monotrait ratio (HTMT).

	CER	CEU	CPD	CWC	KIP	KPS	KSP	KSV	MS	PCI	PFI	PFM	PJC	SFD	SID	SIO	SSS	TAV	TFB	TTI	TTS	TTV	WID	WPP	WTC	WVO
CER																										
CEU	0.92																									
CPD	0.94	0.98																								
CWC	0.95	1.00	1.00																							
KIP	0.64	0.63	0.57	0.63																						
KPS	0.65	0.65	0.58	0.63	1.02																					
KSP	0.61	0.63	0.54	0.60	0.92	0.92																				
KSV	0.73	0.70	0.64	0.69	1.00	1.00	0.96																			
MS	0.78	0.77	0.76	0.76	0.72	0.73	0.69	0.75																		
PCI	0.77	0.77	0.74	0.76	0.76	0.76	0.72	0.78	0.88																	
PFI	0.78	0.78	0.77	0.78	0.73	0.73	0.71	0.78	0.87	0.98																
PFM	0.79	0.78	0.73	0.76	0.80	0.81	0.76	0.84	0.86	0.95	0.97															
PJC	0.77	0.78	0.72	0.76	0.73	0.73	0.71	0.74	0.87	0.96	0.98	0.95														
SFD	0.70	0.77	0.73	0.74	0.61	0.62	0.55	0.60	0.72	0.70	0.68	0.66	0.68													
SID	0.68	0.72	0.71	0.69	0.54	0.53	0.44	0.51	0.70	0.66	0.62	0.59	0.63	0.99												
SIO	0.64	0.67	0.67	0.65	0.55	0.56	0.45	0.53	0.66	0.62	0.57	0.54	0.56	0.91	0.93											
SSS	0.66	0.72	0.69	0.69	0.53	0.54	0.46	0.52	0.71	0.63	0.61	0.60	0.62	1.00	1.00	0.92										
TAV	0.69	0.69	0.66	0.69	0.68	0.67	0.71	0.66	0.75	0.72	0.71	0.75	0.71	0.67	0.58	0.57	0.62									
TFB	0.60	0.55	0.53	0.53	0.58	0.62	0.57	0.61	0.61	0.60	0.56	0.59	0.53	0.64	0.55	0.53	0.62	0.83								
TTI	0.56	0.52	0.47	0.51	0.59	0.61	0.57	0.60	0.65	0.59	0.55	0.62	0.55	0.58	0.48	0.47	0.57	0.81	1.03							
TTS	0.54	0.51	0.46	0.50	0.53	0.55	0.53	0.55	0.60	0.50	0.51	0.55	0.51	0.57	0.47	0.45	0.53	0.74	0.90	0.89						
TTV	0.58	0.54	0.51	0.55	0.58	0.59	0.60	0.57	0.71	0.63	0.57	0.63	0.59	0.62	0.52	0.49	0.59	0.89	0.89	0.92	0.83					
WID	0.80	0.79	0.76	0.79	0.79	0.84	0.75	0.86	0.89	0.90	0.91	0.94	0.90	0.70	0.63	0.58	0.64	0.78	0.64	0.64	0.60	0.69				
WPP	0.80	0.80	0.76	0.78	0.80	0.80	0.75	0.84	0.84	0.95	0.98	0.99	0.95	0.67	0.59	0.56	0.59	0.75	0.60	0.60	0.52	0.61	0.97			
WTC	0.85	0.84	0.80	0.84	0.82	0.83	0.75	0.85	0.92	0.93	0.92	0.93	0.91	0.74	0.69	0.66	0.67	0.76	0.64	0.66	0.59	0.68	1.01	0.97		
WVO	0.82	0.80	0.77	0.78	0.79	0.80	0.74	0.85	0.86	0.91	0.92	0.94	0.91	0.68	0.61	0.57	0.62	0.76	0.62	0.64	0.56	0.63	0.98	0.96	0.98	

Given that it is less skewed and more accurate for second- and third-order construct scores, a repeated indicator technique with model “B" should be used in reflecting formative higher-order latent constructs [45]. Proactive behavior was the third-order endogenous element in the study, whereas task, knowledge, social, and contextual elements were the second-order exogenous constructs.

The development of these reflective-formative second- and third-order structures took into account outer weights, outer loadings, and VIF values [44]. Table 4 shows that the VIF values for all structures were less than 5 [53], which allowed for the detection of co-linearity. The cutoff point was exceeded by the value of the external loads and weights. Therefore, none of the third- or second-order constructs have problems with validity.

**Table 4:** Validating second and third order constructs.

Constructs	Order of constructs	Outer weights	Outer loadings	VIF
CC	Second order	1.000	1.000	1.000
KNC	Second order	1.000	1.000	1.000
SC	Second order	1.000	1.000	1.000
TC	Second order	1.000	1.000	1.000
PAB	Third order	1.000	1.000	1.000

Note. CC= Contextual characteristics; KNC= Knowledge Characteristics; MS= Motivational States; PAB= Proactive Behavior; SC= Social Characteristics and TC= Task Characteristic.

With acceptable composite reliability, convergent validity, and discriminate validity in first, second, and third-order constructs, the suggested conceptual model was therefore expected to be acceptable. So, it is possible to run the structural model.

#### 4.2.2 Structural model

Assessing the structural model is not considered to be the lower-order components when the measurement model assessment is satisfactory [54], and this information is provided using the overall fit of the estimated model, the statistical significance and relevance of the path coefficients, the effect size, the coefficient of determination (R^2^), and the blindfolding-based cross-validated redundancy measure Q2 [50].

##### 4.2.2.1 Overall fit of the estimated model

The overall fit of the predicted model was evaluated by a bootstrap-based test with a standardized root mean square residual (SRMR) Measure. Henseler, Ringle, and Sarstedt [52] created the SRMR as a goodness-of-fit measure for PLS-SEM that can prevent model miss-specification in place of the traditional goodness-of-fit index (GFI). The SRMR value should be less than 0.10 or 0.080, which denotes a good or acceptable model fit, according to a preliminary estimation of the value [55]. The SRMR is a measure of approximate fit used to gather data to back up the idea that has been put out.

The value of the SRMR was 0.045, as shown in Table 5. Which is less than 0.08, and the NFI score was 0.945, which exceeded the criterion of 0.9. This means that the proposed model is well suited to supporting and clarifying the current theory, according to the finding.

**Table 5:** Model fit.

	Saturated Model	Estimated Model
SRMR	0.045	0.045
NFI	0.945	0.945

##### 4.2.2.2 The statistical significance and relevance of the path coefficients

The bootstrapping method was used to evaluate the significance of the hypothesis [55]. To assess the importance of the path coefficient and T-statistics values, the authors employed 5000 sub-samples with no significant changes, 279 cases, and a bootstrapping method.

**Fig. 3:**
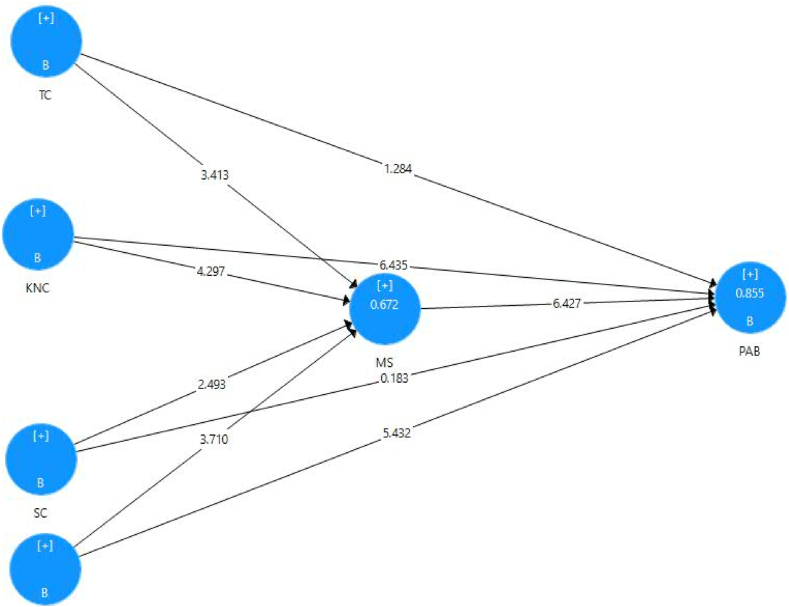
The inner model of PLS- SEM

Table 6 and Fig. 3 both display the structural model of the path coefficients. The correlation coefficients were β= (0.299, 0.259 & 0.383) and t value = (5.493, 6.411 & 6.541 > 1.96), respectively, which showed a strong positive relationship between contextual factors, knowledge characteristics, and proactive behavior. Social and task characteristics don't matter much when it comes to proactive behavior, though. Because of this, Ha2 and Ha4 are accepted but Ha1 and Ha3 are rejected.

**Table 6:** Path coefficients.

	Original Sample (O)	Sample Mean (M)	Standard Deviation (STDEV)	T Statistics (|O/STDEV|)	P Values
CC - > MS	0.313	0.313	0.085	3.663	0.000
CC - > PAB	0.299	0.297	0.054	5.493	0.000
KNC - > MS	0.259	0.260	0.061	4.256	0.000
KNC - > PAB	0.300	0.301	0.047	6.411	0.000
MS - > PAB	0.383	0.382	0.059	6.541	0.000
SC - > MS	0.188	0.191	0.075	2.496	0.013
SC - > PAB	0.008	0.009	0.042	0.191	0.849
TC - > MS	0.206	0.201	0.061	3.352	0.001
TC - > PAB	0.060	0.061	0.047	1.291	0.197

Note. CC= Contextual characteristics; KNC= Knowledge Characteristics; MS= Motivational States; PAB= Proactive Behavior; SC= Social Characteristics and TC= Task Characteristics.

The contextual, knowledge, social, and task characteristics and motivational state are significantly positively correlated, as shown by the values of β = (0.313, 0.259, 0.188 & 0.206) and t value = (3.663, 4.256, 2.496, 3.352 > 1.96), respectively, in the same table. Therefore, Ha5, Ha6, Ha7, and Ha8 are accepted. While the t values of (6.541 > 1.96) and β = (0.383) respectively demonstrated the validity of the link between motivational state and proactive behavior. Therefore, Ha9 is accepted.

##### 4.2.2.3 The effect sizes (f^2^)

Effect size (f^2^) is a metric used by Kumar [49], p. 22] to quantify the influence of each exogenous latent construct on the endogenous latent construct. The values of f^2^ were 0.35 (strong influence), 0.15 (moderate effect), and 0.02 (weak effect), in agreement with Cohen's [56] recommendations. As a result, Table 7 demonstrates that the impact sizes for (f^2^), CC, KNC, and MS on PAB were 0.201, 0.261, and 0.299, respectively. The model is accurate and the constructs are significant for the overall change of the model, as evidenced by the values being greater than 0.15 and having a considerable effect. PAB is only marginally impacted by TC, and SC has no effect.

**Table 7:** f Square.

	CC	KNC	MS	PAB	SC	TC	
Contextual characteristics (CC)			0.118	0.201			
Knowledge Characteristics (KNC)			0.102	0.261			
Motivational States (MS)				0.299			
Proactive Behavior (PAB)							
Social Characteristics (SC)			0.056	0.002			
Task Characteristics (TC)			0.062	0.010			

##### 4.2.2.4 Coefficient of determination (R^2^)

R^2^ is a measurement of a model's prediction accuracy [55]. A “rough” generalization of large, moderate, or poor levels of predictive accuracy is indicated by an acceptable R^2^ of 0.75, 0.50, and 0.25, respectively [52]. Jr, Matthews, Matthews, and Magdeburg [57] respond that the estimated size of R^2^ depends on the phenomenon being examined. For instance, Hair, Risher, Sarstedt, and Ringle [58] noted that higher than 0.90 R^2^ values in a model that predicts human attitudes, perceptions, and intentions could likely demonstrate over fit whereas higher R^2^ is connected with more predictor components. Five components were used as predictors in this study, and the R^2^ value was less than 0.9.

Table 8 shows that the coefficient of determination, adjusted R^2^, is 0.853 for the proactive behavior and 0.666 for the motivational state as an endogenous latent construct. This means that the five latent constructs (task characteristics, knowledge characteristics, social characteristics, contextual characteristics, and motivational state) substantially explain 85.3% of the variance in PAB while, task, knowledge, social and contextual characteristics collectively explain 66.6% of the variance of motivational state.

**Table 8:** Coefficients of determination.

	R Square	R Square Adjusted
Motivational State (MS)	0.671	0.666
Proactive Behaviour (PAB)	0.855	0.853

##### 4.2.2.5 Cross-validated redundancy (Q^2^)

The path model has a predictive value for any endogenous construct for which Q2 has a value greater than zero [55]. Using Q^2^, you may assess the inner model's prediction usefulness. The Q^2^ values for two endogenous constructs (MS and PAB) were shown in Table 9 to be 0.441 and 0.826, respectively, and to be greater than zero. As a result, it can be concluded that the model is accurate and that the constructs are essential to the overall change of the model.

**Table 9:** Construct cross validated redundancy.

	SSO	SSE	Q^2^ (=1-SSE/SSO)
Contextual Characteristics (CC)	279	279	
Knowledge Characteristics (KNC)	279	279	
Motivational State (MS)	1674	936.506	0.441
Proactive Behavior (PAB)	279	48.56	0.826
Social Characteristics (SC)	279	279	
Task Characteristics (TC)	279	279	

### 4.3 The mediating effect of motivational state

In order to study the mediating influence of the motivational state on the link between work characteristics (task, knowledge, social, and contextual features), and proactive behavior, the authors utilized a Bootstrapping analysis to determine the PLS-SEM means and standard deviations [46]. The relationship between the exogenous and endogenous latent variables is either fully mediating or partially mediating, as seen by the coefficient and t-statistics. The mediation of endogenous latent factors was partially and fully justified by the direct and indirect effects of exogenous latent variables.

As demonstrated in Table 10 and Fig. 3, hypothesis testing based on the PLS-SEM link among the latent components suggests the direct and indirect effects of contextual, knowledge, social, and task aspects on proactive behavior. The path coefficient in SMART PLS can be used to explain how exogenous latent constructs (TC, KNC, SC, and CC) affect endogenous latent construct (PAB) when a mediator (MS) is present. The results show a direct relationship between contextual and knowledge aspects and proactive behavior, with t values of (5.493 & 6.411 > 1.96) and β = (0.299 & 0.300) correspondingly. However, task and social characteristics do not significantly and positively influence proactive behavior (see Table 10).

**Table 10:** Direct and indirect effect.

Constructs	Original Sample (O)	Sample Mean (M)	Standard Deviation (STDEV)	T Statistics (|O/STDEV|)	P Values
Direct effect					
CC - > PAB	0.299	0.297	0.054	5.493	0.000
KNC - > PAB	0.300	0.301	0.047	6.411	0.000
SC - > PAB	0.008	0.009	0.042	0.191	0.849
TC - > PAB	0.060	0.061	0.047	1.291	0.197
Indirect effect					
CC - > MS - > PAB	0.120	0.121	.039	3.069	.002
KNC - > MS - > PAB	0.099	.099	.028	3.586	.000
SC - > MS - > PAB	0.072	.072	.030	2.440	.015
TC - > MS - > PAB	0.079	.078	.027	2.875	.004

Note. CC= Contextual characteristics; KNC= Knowledge Characteristics; MS= Motivational States; PAB= Proactive Behavior; SC= Social Characteristics and TC= Task Characteristics.

According to the findings of a specific indirect effect, all four exogenous latent variables (CC, KNC, SC, and TC) significantly influence proactive behavior through motivational state, with t values of 3.069, 3.586, 2.440, and 2.875 > 1.96, respectively. Therefore, Ha10, Ha11, Ha12, and Ha13 were accepted. For CC, KNC, SC, and TC, respectively, there is a standardized indirect relationship between employee motivational state and PAB of 12.10%, 9.9%, 7.2%, and 7.9%. This suggests that while assuming that all other model variables remain constant, the standard deviations of PAB will increase by 0.039, 0.028, 0.030, and 0.027 from its mean if the impact of CC, KNC, SC, and TC on PAB through MS increases one standard deviation from its mean.

Based on their findings, the researchers distinguished between direct and indirect influences, as well as between partial and full mediation of the motivational state. Therefore, the link between the exogenous latent constructs (task, knowledge, and contextual factors) and the endogenous latent construct (proactive behavior) is partially mediated by the motivational state. As a result, both the direct effect and the indirect effect are significant.

Because the direct influence is negligible while the indirect effect is significant, social characteristics and proactive behavior are fully mediated. Table 11 shows that whereas task, knowledge, and contextual qualities considerably positively influence proactive behavior, social characteristics have no overall significant influence. Therefore, social traits and proactive behavior have not been related in the absence of the motivational state's mediation function.

**Table 11:** Total effect.

	Original Sample (O)	Sample Mean (M)	Standard Deviation (STDEV)	T Statistics (|O/STDEV|)	P Values
CC - > MS	0.312	0.314	0.085	3.695	0.000
CC - > PAB	0.418	0.420	0.074	5.630	0.000
KNC - > MS	0.259	0.258	0.061	4.266	0.000
KNC - > PAB	0.399	0.397	0.051	7.765	0.000
MS - > PAB	0.383	0.383	0.059	6.448	0.000
SC - > MS	0.189	0.190	0.074	2.560	0.010
SC - > PAB	0.080	0.080	0.056	1.419	0.156
TC - > MS	0.206	0.203	0.062	3.311	0.001
TC - > PAB	0.139	0.139	0.046	3.037	0.002

Note. CC= Contextual characteristics; KNC= Knowledge Characteristics; MS= Motivational States; PAB= Proactive Behavior; SC= Social Characteristics and TC= Task Characteristics.

## 5 Discussions

The authors highlighted the mediating role of the motivational state as they briefly investigated the association between the four work characteristics and proactive behavior in light of the study's findings. Theoretically, work design theory and research are evolving [1]. Empirical research has shown that workplace features, such as task, knowledge, social, and contextual characteristics, have an impact on proactive behavior [29]. Therefore, the writers backed up these theoretical and empirical findings.

Our results indicate a positive, significant association between proactive behavior and task characteristics. To be more specific, job autonomy, task variety, task relevance, task identity, and job feedback are all factors that positively predict proactive behavior. Despite the fact that proactive behavior is initiated by employees rather than prescribed or directed at them by others, task characteristics are a better approach to assess an employee's proactivity. Change should need self-initiated and future-focused behaviors, as shown by the well-established task characteristics prevalent in the workplace [24,26]. The findings of this study provide evidence in support of earlier theoretical hypotheses and empirical findings (for example, those in Refs. [18,24,and32]]).

Higher educated employees who can handle problem-solving, a diversity of talents and specialization are more likely to take the initiative. Knowing more about the underlying mechanisms will help you comprehend the phenomena of proactive behavior since knowledge is the basis of employee proactive behavior [4,27]. Knowledgeable employee behavior was positively connected with knowledge attributes, according to Dust and Resick [29]. Given the strong positive correlation between knowledge characteristics and proactive behavior in our data, these ideas are supported.

Additionally, proactive behavior and contextual factors have a significant positive link, according to the study's findings. Proactive behavior has its roots in the ergonomics, physical requirements, working conditions, and equipment that are specific to the environment. Our findings and those of other studies like [4,14] are consistent.

Positive significant relationships between social characteristics and proactive behavior did not exist without the mediation role of the motivational state (energized to). Studies have not agreed on how these constructions relate to one another directly or indirectly in this regard. Some researchers observed a link between social traits and proactive behavior that was indirect [5,49,50], whereas others discovered a link directly between social traits and the qualities of an autonomy task [30]. Our findings demonstrate that social qualities and proactive behavior have inverse relationships thanks to the energized to state's function as a mediator. According to Cai, Parker, Chen, and Lam's meta-analysis [5], social context elements have a significant impact on employees' “reason to,” “can do,” and “energized to” states, which in turn affect how proactive they are.

The results of our study show that proactive behavior and a motivational state are strongly correlated. Positive affect (energy), according to prior studies, is more likely to motivate staff to take initiative [18,50,51]. A prominent mechanism that has garnered interest [34] is the energized to state (positive affect), which supports proactive behaviors [4]. When in a positive affective state, employees are more likely to set challenging goals and take decisive action to achieve those goals [59].

Parker, Wang, and Liao's research [59] indicates that the motivational state provides a useful theoretical framework for examining mediating elements that might link work characteristics with proactive behaviour. The energized state (positive affect) will also play a greater role in influencing employees' behaviour, according to Wang, Xu, Sun, and Liu's hypothesis [37]. Schmitt, and Belschak [18] looked explored how the motivational states of “reason to,” “energized to,” and “can do” can serve as the building blocks of proactive behaviour in the workplace (task, knowledge, social, and contextual factors). In contrast to task, knowledge, and contextual factors, which are only marginally mediated by proactive behaviour, our research shows that the motivational state fully mediates the association between social traits and proactive behavior.

## 6 Conclusion

This study intends to examine the link between task, knowledge, social, and environmental work-related characteristics and proactive behavior by employing a motivational state as a mediating component. In order to achieve this purpose, the authors looked at the evidence and reached their conclusions. Firstly, the factors that were most likely to be linked to proactive activity in employees were task, knowledge, contextual, and motivational factors; social qualities and proactive behaviour, however, had a more tenuous connection. The four work-related characteristics are closely linked to the energized state of mind. Thirdly, the motivated state partially mediates the association between work-related traits (such as task, knowledge, and contextual aspects) and proactive behavior while mediating totally the relationship between social characteristics and proactive behaviour. The authors emphasise the indirect relationship between social characteristics and proactive behaviour through motivational state and call for more research to support their findings and advance their model. More specifically, they want to know how proactive behaviour affects social characteristics. Additionally, a stronger emphasis was placed on the mediating role of motivational state.

The findings of this study will assist managers in the workplace with the management of proactive processes and behaviours. Because proactive behaviour is self-directed and future-focused, organizations benefit from proactive individuals who drive improvements at work (3). Policies may be created for the success of the current economic transition by considering the relationship between work features and proactive behaviour, as well as the consequences for employee proactivity and industrial parks. In order to promote proactive behaviour among staff members and to emphasise the task, knowledge, and context-specific components of their work, managers of organizations must play a critical role. They can also make use of their enthusiasm to motivate staff members' efforts.

## 7 Limitation of the study

Although writers incorporated self-report and supervisory measures and obtained data from workers who had immediate supervisors, several limitations have been observed. Since a cross-sectional survey was used to collect all of the data, some of our conclusions may have been affected by common-method biases. Taking into account that the authors sought to collect data from the industrial parks at Hawassa, Bole leme, and Combolcha. However, we decided to gather our data from Debrebirhan Industrial Park instead of Combolcha due to the disagreement that erupted during the time of data collection and may have affected our sample. In order to ascertain the mean difference in the results, future research should make an effort to update this study by analysing a self report and supervisory measures separately. The fact that we focused on operational staff and industrial parks in the garment industry may have restricted the applicability of our findings. Follow-up research, though, should support our findings by examining all industrial park sectors and using larger employee samples.

## Funding

This research did not receive any specific grant from funding agencies in the public, commercial, or not-for-profit sectors.

## Production notes

### Author contribution statement

Woretaw Chanie: Conceived and designed the experiments; Performed the experiments; Analyzed and interpreted the data; Wrote the paper.

Selomon Ambaw; Assegid Demissie: Contributed reagents, materials, analysis tools or data; Wrote the paper.

### Data availability statement

Data will be made available on request.

## Declaration of competing interest

The authors declare that they have no known competing financial interests or personal relationships that could have appeared to influence the work reported in this paper.
